# The reliability of SARS-CoV-2 IgG antibody testing – a pilot study in asymptomatic health care workers in a Croatian university hospital

**DOI:** 10.3325/cmj.2020.61.485

**Published:** 2020-12

**Authors:** Ivana Lapić, Dunja Rogić, Dragana Šegulja, Saša Kralik Oguić, Josip Knežević

**Affiliations:** Department of Laboratory Diagnostics, University Hospital Center Zagreb, Zagreb University School of Medicine, Zagreb, Croatia

## Abstract

**Aim:**

To evaluate three fully automated serological assays in terms of reactivity to SARS-CoV-2 immunoglobulin G (IgG) and perform SARS-CoV-2 IgG antibody testing among asymptomatic health care workers (HCW) at the University Hospital Center Zagreb.

**Methods:**

Three IgG serological assays (Abbott SARS-CoV-2 IgG, Elecsys Anti-SARS-CoV-2, and MAGLUMI 2019-nCoV IgG) were initially evaluated by analyzing 42 samples from confirmed COVID-19-recovered patients and 48 negative individuals. A total of 1678 HCW ( ~ 30% of all hospital employees) were screened for SARS-CoV-2 IgG with the Abbott assay, run on Abbott Architect i2000SR. The samples exceeding the predefined cut-off (1.4 S/C) were reanalyzed with the Elecsys, MAGLUMI, and VIDAS SARS-COV-2 IgG assays.

**Results:**

Initially, the MAGLUMI 2019-nCoV IgG produced 26.2% false negatives and the Elecsys Anti-SARS-CoV-2 produced one false positive. Among 1678 HCW, the Abbott assay showed only 10 (0.6%) positive results, with mostly mildly elevated signals. Nine of these samples were non-reactive when they were retested with the Elecsys, MAGLUMI, and VIDAS assays. As for the one remaining sample, it was positive when tested with the Elecsys assay, while the other two assays yielded negative results.

**Conclusions:**

SARS-CoV-2 IgG seroprevalence among asymptomatic HCW in our hospital setting was low, with different assays indicating a different number of positive samples. One of the assays yielded a large false negative rate. These findings can be attributed to differences in assay formulation but also to heterogeneity and diverse reactivity of antibodies against SARS-CoV-2 antigens.

At the beginning of the coronavirus disease 2019 (COVID-19) pandemic, health care institutions needed to quickly establish organizational changes in order to be able to diagnose and treat COVID-19, and suppress the virus spread (1,2). COVID-19 poses an especially high risk for health care workers (HCW) as they are directly exposed to potential virus contamination (3), even if following obligatory biosafety practices, including social distancing measures, wearing protective equipment, enhanced personal hygiene, and surface disinfection protocols (4). COVID-19 infections among HCW lead to long-term work absences and staff shortages, and place a significant additional burden on the already overwhelmed health care system (1). Moreover, a high prevalence of asymptomatic or mildly symptomatic COVID-19 patients, together with long incubation of up to 14 days, may result in disease underdiagnosis and increased HCW-mediated viral transmission (1,4,5).

Since HCW are at the frontline of the COVID-19 pandemic, it is necessary to assess the true extent of viral contagion in this group of professionals. The gold standard for the diagnosis of acute COVID-19 infection is viral identification in nasopharingeal and/or oropharyngeal swab specimens with reverse transcription real-time polymerase chain reaction (rRT-PCR). However, serology testing serves as a complementary, non-invasive diagnostic tool for detecting antibody response to SARS-CoV-2, identifying asymptomatic carriers and tracking seroconversion (6-8). Following COVID-19 infection, nearly all immunocompetent individuals develop an antibody response (9). Immunoglobulin M antibodies specific for SARS-CoV-2 antigens are detectable within seven days from symptoms onset, while immunoglobulin G (IgG) antibodies are detectable shortly afterwards, although the exact dynamics of immunological response is still largely unknown and might vary considerably among individuals (7,10-13). The presence of neutralizing IgG points to late-term immunity and might prevent re-infection (11,14), meaning a safe return of HCW to the workplace and the maintenance of appropriate staffing during the pandemic.

Therefore, a month after the beginning of the pandemic in Croatia, we conducted a large scale serology testing within our institution with the aim to determine the seroprevalence of SARS-CoV-2 IgG antibodies among HCW and assess the proportion of infected asymptomatic HCW.

## Participants and methods

### Study setting

The study was performed at the Department of Laboratory Diagnostics, University Hospital Center (UHC) Zagreb, Croatia. UHC Zagreb is the largest tertiary academic hospital in Croatia, consisting of 28 medical departments with 1800 beds and employing 5500 people (about 80% of whom are health care professionals). Although the hospital is not a dedicated COVID-19 hospital, at the beginning of the pandemic it underwent massive organizational changes to adapt to a sharply increasing number of emergency patients who would normally have been distributed between two hospitals. In order to suppress the virus spread, the hospital introduced strict preventive measures, including thorough patient triage, obligatory wearing of face masks, body temperature measurements, and hand disinfection at entrance points. Moreover, all hospital staff strictly adhered to social distancing, wore personal protective equipment, and implemented preventive hygiene measures.

### Serological assays

The serology testing in this study was performed with four serological assays.

1) The SARS-CoV-2 IgG (Abbott Laboratories, Abbott Park, IL, USA) is a qualitative fully automated chemiluminescent immunoassay run on Abbott Architect i2000SR immunoassay analyzer (Abbott Laboratories, Abbott Park, IL, USA). It detects the presence of IgG antibodies to the nucleocapside protein of SARS-CoV-2 in the serum or plasma. The result is expressed in relative light units (RLU) and reported as an index calculated by dividing the signal of the tested sample by the calibrator (sample/calibrator, S/C). The cut-off is 1.4 S/C. According to the manufacturer, the assay has a diagnostic sensitivity at ≥14 days after symptom onset of 100% and diagnostic specificity of 99.6% (15).

2) The Elecsys Anti-SARS-CoV-2 is a fully automated qualitative electrochemiluminescent immunoassay run on Cobas 6000 analyzer series (both from Roche Diagnostics, Mannheim, Germany). It detects the presence of total antibodies (including IgG) against SARS-CoV-2 using a recombinant protein representing its nucleocapside antigen. The result is expressed as a cut-off index (COI), calculated by dividing the electrochemiluminescence signal of the sample with the signal obtained by calibration. The cut-off is 1.0 COI. According to the manufacturer (16), the assay has a diagnostic sensitivity of 100% at least 14 days after rRT-PCR confirmation and a specificity of 99.8%.

3) The MAGLUMI 2019-nCoV IgG is a fully automated chemiluminescent immunoassay run on MAGLUMI^TM^ 800 immunoassay analyzer (both from Shenzhen New Industries Biomedical Engineering Co., Ltd [Snibe], Shenzhen, China). It uses antibodies directed against both the spike and viral nucleocapside SARS-CoV-2 protein (12). The cut-off is 1.0 arbitrary units per milliliter (AU/mL). According to the manufacturer, the assay has a diagnostic sensitivity and specificity of 91.2% and 97.3%, respectively (17).

4) The VIDAS^®^ SARS-COV-2 IgG assay is a semi-automated qualitative assay run on miniVidas analyzer (both from bioMérieux, Marcy-l'Étoile, France). It is based on the enzyme-linked immunofluorescent principle and detects IgG specific for SARS-CoV-2 nucleocapside and spike protein. The result obtained in relative fluorescence values (RFV) is divided by the RFV of the provided standard. The cut-off is 1.0 RFV. Assay sensitivity is 96.6% at ≥16 days after positive rRT-PCR confirmation (18).

The reactivity of the Abbott, Elecsys, and MAGLUMI assays toward SARS-CoV-2 IgG was preliminary evaluated by analyzing a series of 42 samples obtained from recovered COVID-19 patients, in whom the infection was confirmed by rRT-PCR at least 30 days ago. Additionally, we tested 48 presumably negative, healthy volunteers who strictly followed all preventive measures and were not in contact with either COVID-19 patients or anyone outside their own household. The false negative (FN) and false positive (FP) rates were calculated. This part of the study did not involve the use of the VIDAS SARS-COV-2 IgG assay.

Given the different assay formulation and reactivity toward target antigens, we also analyzed non-proprietary control samples, ie, SARS-CoV-2 IgG-positive and negative commercial control samples with the Elecsys and MAGLUMI assay, and MAGLUMI 2019-nCoV IgG-positive and negative control samples with the Abbott and Elecsys assay. At the time of conducting this study, dedicated commercial control materials for Elecsys Anti-SARS-CoV-2 were not available.

### Serology testing of asymptomatic health care workers

The study enrolled 1678 asymptomatic HCW (median age, 43 years) who were actively involved in patient care and associated hospital activities from mid-March to the end of April 2020. All asymptomatic HCW with previously confirmed COVID-19 were excluded from this part of the study.

All recruited HCW were initially tested with the Abbott SARS-CoV-2 IgG assay. The positive samples were reanalyzed with three other serological assays.

The samples were collected during May 2020. From each participant, one 5-mL serum tube (Becton Dickinson, Wokingham, United Kingdom) was obtained. The analyses were performed on fresh samples after centrifugation within 8 hours from blood draw. The study was approved by the University Hospital Center Zagreb Ethics Committee, and all participants gave informed consent before enrollment.

## Results

### Evaluation of serological assays

The Abbott assay initially yielded no false positives or false negatives, the Elecsys yielded one false-positive result, while the MAGLUMI obtained 11 false negatives (26.2%) (Table 1).

**Table 1:** The analysis of samples from confirmed coronavirus disease 2019 (COVID-19) patients and negative individuals with the Abbott SARS CoV-2 IgG, Elecsys Anti-SARS-CoV-2, and MAGLUMI 2019-nCoV IgG assays*

			Confirmed COVID-19 patients (N = 42)		COVID-19-negative individuals (N = 48)
Assay	Manufacturer	Cut-off	median (IQR)	positive /negative (% false negative)	positive/negative (% false positive)
SARS-CoV-2 IgG	Abbott Laboratories, Abbott Park, IL, USA	1.4 S/C	6.0 (4.5-7.8)	42/0 (0)	0/48 (0)
Elecsys Anti-SARS-CoV-2	Roche Diagnostics, Mannheim, Germany	1.0 COI	81.3 (25.0-123.1)	42/0 (0)	1/47 (2.1)
MAGLUMI 2019-nCoV IgG	Shenzhen New Industries Biomedical Engineering Co., Ltd (Snibe), Shenzhen, China	1.0 AU/mL	3.9 (1.0-11.8)	31/11 (26.2)	0/48 (0)

*S/C – sample/calibrator ratio; COI – cut-off index; AU/mL – arbitrary units per milliliter; IQR – interquartile range.

Neither the Abbott nor the Elecsys assay obtained any signal in negative and positive MAGLUMI control material. On the contrary, in negative and positive Abbott control material the MAGLUMI and Elecsys assay obtained signals concordant with their declared reactivity, ie, negative or positive.

### Testing of asymptomatic health care workers

Abbott SARS-CoV-2 IgG testing was performed in 1678 participants ( ~ 30% of all hospital employees), 1312 (78.2%) of whom were female. The greatest number of positive individuals worked at the Department of Gynecology and Obstetrics (16.4%), followed by the Department of Anesthesiology and Intensive Care Unit (13.2%) and Department of Internal Medicine (10.7%) (Supplementary Table 1(supplementary Table 1)).

The majority of study participants were health care personnel (89.5%), while the rest worked in hospital administrative and support, technical support, and cleaning services (Supplementary Table 2(supplementary Table 2)).

The Abbott SARS-CoV-2 IgG assay yielded only 10 (0.6%) positive results. Nine of these samples were non-reactive when reanalyzed with the Elecsys Anti-SARS-CoV-2, MAGLUMI 2019-nCoV IgG, and VIDAS SARS-COV-2 IgG assays. The median S/C value of these nine samples obtained with the Abbott SARS-CoV-2 IgG assay was 1.71 (interquartile range: 1.68-2.73). The remaining one sample was classified as positive when reanalyzed with the Abbott assay (S/C value of 8.75) and Elecsys Anti-SARS-CoV-2 (2.06 COI), while the other two assays produced negative results.

## Discussion

The present study revealed a low SARS-CoV-2 IgG seroprevalence in a large representative sample of asymptomatic HCW from various hospital departments at the UHC Zagreb. This is a hardly surprising finding since a relatively low prevalence among asymptomatic HCW was observed in even more burdened settings, such as referral hospitals in Germany, Spain and United Kingdom, had (1.6%, 1.9%, and 3%, respectively) (19-21). Furthermore, immediately after COVID-19 outbreak in Croatia, our hospital introduced a number of organizational and strategic changes. The changes included immediate implementation of rRT-PCR testing, obligatory patient triage, testing of all suspected patients before hospital admission, isolation of suspected patients awaiting laboratory confirmation in a COVID-19 segregated part of the Emergency Department, and prompt transfer of diagnosed patients to a COVID-19 dedicated hospital. Staff splitting, effective contact tracing, and quarantines additionally contributed to limiting HWC-mediated virus spread. Finally, an important factor that could explain the low seroprevalence observed in this study is the overall low incidence of confirmed COVID-19 cases in Croatia in the investigated period (ie, 2246 per 4.08 million inhabitants, as of June 1, 2020).

Although seroprevalence among HCW in our setting was very low, different seroprevalence rates obtained with different serological assays indicate that at this point the exact number of positive HCW cannot be determined. The currently available serological assays differ significantly in terms of the targeted SARS-CoV-2 antigen, assay format, and signal generation, with diverse reactivity and capture specificity of individually produced antibodies against SARS-CoV-2 antigens (22-24). These differences were confirmed by our initial serological screening with the Abbott SARS-CoV-2 IgG. The screening revealed 10 positive samples (0.6%), nine of which were subsequently classified as negative by other assays. It is important to note that these nine samples mostly yielded mildly elevated signals. This finding clearly deserves further investigation and calls for reconsideration of the threshold proposed by the manufacturer (22). In addition, possible analytical interferences should be carefully evaluated. These mainly include cross-reactivity with antibodies to the common circulating coronaviruses and different autoimmune diseases or past viral infections, especially cytomegalovirus, which was reported by the manufacturer to cause FP results (13,22).

Our preliminary evaluation revealed occasional discrepancies in assay reactivity, probably because of differences in assays' formulation or antigen presentation. This was especially evident in the case of the MAGLUMI nCoV-IgG, which yielded a striking FN rate. The MAGLUMI nCoV-IgG and VIDAS SARS-COV-2 IgG also classified one asymptomatic HCW as negative, while the other two assays classified him or her as positive, which raised concerns that this might be another true positive. However, the results obtained were not linear when the sample was serially diluted, which is not the case with true positives. This discordance might be explained by the fact that the Abbott and Elecsys assays detect antibodies directed to the nucleocapside, while the MAGLUMI and VIDAS assays detect antibodies against both the spike protein and nucleocapside. Moreover, different target antigens might at least partly explain the fact that we obtained no signal when the MAGLUMI-negative and -positive controls were analyzed with the Abbott and Elecsys assays but obtained signals concordant with their declared reactivity when Abbott controls were analyzed with the MAGLUMI and Elecsys assay. However, specific control constitution, non-commutability of commercial control samples, and matrix-dependent reactivity of each immunoassay could have also contributed to these findings. It is noteworthy that all available assays, although being declared as qualitative, provide a kind of quantitative signal relative to a fixed calibrator value, with intensities largely dependent on assay composition and reaction kinetics. This makes it difficult to compare the quantitation of results obtained by different serological assays (23). Given all this, interchangeable use of different serological assays is not advisable, for either seroprevalence studies or longitudinal patient monitoring.

Our study has some limitations. First, owing to limited resources, all study participants were initially screened only with Abbott SARS-CoV-2 IgG assay. Second, only a short initial assessment of assays' performance was performed, not including the VIDAS SARS-COV-2, due to limited reagent quantity. Finally, the positive results were not tested with a reliably validated confirmation method, such as virus neutralization test. However, in an effort to minimize the number of FP results, a testing algorithm with alternative assays was applied, as recommended by the Center for Disease Control and Prevention (25).

In conclusion, this study points to a negligible SARS-CoV-2 IgG seroprevalence among asymptomatic HCW in our hospital setting, which indicates that similarly low rate of acquired immunity was present among the general population in Croatia in the observed period. Although conducted in a non-COVID-19 designated hospital with only occasionally confirmed COVID-19 infections, our study provides valuable results for epidemiological surveillance that became pivotal during the second pandemic wave. While wide serological testing is nowadays strongly advocated, and the laboratory market is quickly becoming overwhelmed with diverse serological assays, scarce peer-reviewed data are available about their diagnostic performance in clinical settings. Thus, our knowledge stems predominantly from manufacturers' data, which seem to be overly optimistic. These data are derived from studies limited by sample size, variable sampling time in relation to symptoms onset, and different patients' characteristics, which results in possible spectrum bias and overestimated diagnostic accuracy. Low-prevalence settings such as ours are especially challenging and require the use of assays with excellent diagnostic specificity in order to avoid FP results. Thus, the existing antibody assays should be carefully evaluated in different disease prevalence settings and applied with caution (13,26,27). Our results point to the substantial possibility of FP findings in low-prevalence populations, as well as to the alarming rate of FN results obtained with the MAGLUMI nCoV-IgG.
