# Supplementary material for: The reliability of SARS-CoV-2 IgG antibody testing – a pilot study in asymptomatic health care workers in a Croatian university hospital
Source: Croat Med J. 2020 Dec;61(6):485–90. doi: 10.3325/cmj.2020.61.485 (PMC7821371; doi:10.3325/cmj.2020.61.485)
Supplement: Supplementary Table 1 [file CroatMedJ_61_s007.pdf]

Supplementary table 1. Distribution of study participants according to hospital department (N=1678)

Hospital department/ward	N (%)
Department of Gynecology and Obstetrics	276 (16.4)
Department of Anesthesiology and Intensive Care Unit	221 (13.2)
Department of Internal Medicine	179 (10.7)
Division of Gastroenterology and Hepatology	62
Internal Intensive Care Unit	58
Division of Nephrology, Hypertension, Dialysis and Transplantation	43
Division of Clinical Immunology and Rheumatology	16
Department of Laboratory Diagnostics	164 (9.8)
Department of Cardiovascular Diseases	158 (9.4)
Department of Oncology and Radiotherapy	119 (7.1)
Department of Emergency Medicine	99 (5.9)

Department of Otolaryngology, Head and Neck Surgery	93 (5.5)
Department of Neurology	38 (2.3)
Department for Respiratory Diseases	35 (2.1)
Department of Orthopedic Surgery	30 (1.8)
Department of Dental Medicine	15 (0.9)
Department of Rheumathology and Rehabilitation	15 (0.9)
Other departments*	61 (3.6)
Hospital administration, technical support, support staff and cleaning services staff	176 (10.4)

*Other departments included in the study in minor proportion (N): Department of Clinical Transfusiology (14), Department of Pathology and Cytology (11), Department of Surgery (10), Department of Dermatovenerology (9), Department of Radiology (8), Department of Urology (5), Department of Pediatrics (4).
