# Supplementary material for: The reliability of SARS-CoV-2 IgG antibody testing – a pilot study in asymptomatic health care workers in a Croatian university hospital
Source: Croat Med J. 2020 Dec;61(6):485–90. doi: 10.3325/cmj.2020.61.485 (PMC7821371; doi:10.3325/cmj.2020.61.485)
Supplement: Supplementary Table 2 [file CroatMedJ_61_s008.pdf]

Supplementary table 2. Distribution of hospital personnel included in the study per working position

Healthcare staff participants<br>-	N (%)
- Nurses and nursing assistants	801 (47.8)
- Medical doctors	366 (21.8)
- Laboratory technicians	124 (7.4)
- Midwives	76 (4.6)
- Medical biochemists and biologists	52 (3.1)
- Radiologic technicians	51 (3.0)
- Physiotherapists	17 (1.0)
- Dentists and dental assistants	15 (0.9)
Non-healthcare staff participants<br>-	
- Cleaning staff	64 (3.8)
- Support staff	51 (3.0)
- Administrative staff	49 (2.9)
- Technical support and security staff	12 (0.7)
